# Circadian and dark-pulse activation of orexin/hypocretin neurons

**DOI:** 10.1186/1756-6606-1-19

**Published:** 2008-12-03

**Authors:** Oliver J Marston, Rhîannan H Williams, Maria M Canal, Rayna E Samuels, Neil Upton, Hugh D Piggins

**Affiliations:** 1 Faculty of Life Sciences, University of Manchester, Manchester, UK; 2 Neurology & GI-CEDD, GlaxoSmithKline Pharmaceuticals, New Frontiers Science Park, Third Avenue, Harlow, Essex ,CM19 5AW, UK

## Abstract

Temporal control of brain and behavioral states emerges as a consequence of the interaction between circadian and homeostatic neural circuits. This interaction permits the daily rhythm of sleep and wake, regulated in parallel by circadian cues originating from the suprachiasmatic nuclei (SCN) and arousal-promoting signals arising from the orexin-containing neurons in the tuberal hypothalamus (TH). Intriguingly, the SCN circadian clock can be reset by arousal-promoting stimuli while activation of orexin/hypocretin neurons is believed to be under circadian control, suggesting the existence of a reciprocal relationship. Unfortunately, since orexin neurons are themselves activated by locomotor promoting cues, it is unclear how these two systems interact to regulate behavioral rhythms. Here mice were placed in conditions of constant light, which suppressed locomotor activity, but also revealed a highly pronounced circadian pattern in orexin neuronal activation. Significantly, activation of orexin neurons in the medial and lateral TH occurred prior to the onset of sustained wheel-running activity. Moreover, exposure to a 6 h dark pulse during the subjective day, a stimulus that promotes arousal and phase advances behavioral rhythms, activated neurons in the medial and lateral TH including those containing orexin. Concurrently, this stimulus suppressed SCN activity while activating cells in the median raphe. In contrast, dark pulse exposure during the subjective night did not reset SCN-controlled behavioral rhythms and caused a transient suppression of neuronal activation in the TH. Collectively these results demonstrate, for the first time, pronounced circadian control of orexin neuron activation and implicate recruitment of orexin cells in dark pulse resetting of the SCN circadian clock.

## Background

The mammalian hypothalamus plays a fundamental role in the control of numerous critical brain and behavior states. For example, within the suprachiasmatic nuclei (SCN), site of the brain's dominant circadian clock, neurons orchestrate daily rhythms responsible for a wide range of physiological and neural processes [1,2]. This SCN circadian clock is synchronized (entrained) by exogenous time cues (zeitgebers) such as varying levels of environmental light (photic stimuli), as well as by so-called non-photic cues that promote behavioral arousal [3,4]. Photic information is conveyed to the SCN via the retinohypothalamic tract [5,6], while non-photic stimuli activate pathways originating in the thalamic intergeniculate leaflet (IGL) and the median raphe (MRN) of the brain stem [7,8]. Unfortunately, our understanding of how neural substrates integrate circadian and arousal information is far from complete.

Recently, significant progress was made in identifying the neurochemical basis of arousal with the isolation and characterization of the orexin/hypocretin neuropeptides (referred to as orexins in this study) [9,10]. There are two bioactive forms, orexin-A (OXA) and orexin-B, which are cleaved from the common precursor prepro-orexin. The orexins are mainly excitatory and mediate their biological actions through two G-protein coupled receptors, OXR1 and OXR2. In mammals, neurons expressing orexins are mostly limited to the lateral hypothalamic area and dorsomedial nucleus of the tuberal hypothalamus (TH). Immunohistochemical studies have established that these orexin-containing neurons have extensive neuronal projections and innervate key circadian structures including the SCN, IGL and MRN [11-13].

SCN efferents project to the TH [14] and may directly contact orexin-containing cells [15]. Transgenic and pharmacological impairment of orexin-OXR1/R2 signaling implicates this neuropeptide system in the promotion of arousal and wakefulness [16]. Indeed, orexin neurons demonstrate diurnal variation in activity/activation, with highest levels in nocturnal animals occurring during the night [17,18]. Since orexin neuronal activation corresponds with forward locomotor activity [19,20], and because such behavior increases at night, it is unclear whether SCN-derived signals regulate the nighttime activation of orexin neurons.

In this study, using c-Fos as a marker of cellular activation, we demonstrate that under constant light (LL), a condition that suppresses the amplitude of wheel-running rhythms, TH cells including OXA-immunoreactive (OXA-ir) neurons are much more active during the subjective night than the subjective day. We also show that activation of OXA-ir neurons in the medial TH occurs prior to the nocturnal onset of vigorous wheel-running. Transient exposure to 6 h of darkness during the subjective day promotes arousal, suppresses c-Fos expression in the SCN and activates OXA-ir neurons, particularly those in the medial TH. Such changes in arousal and cellular activity accompany the resetting actions of this dark pulse stimulus at this time. These results implicate SCN-derived signals in exerting temporal control over TH cells including OXA-ir neurons and highlight extra-SCN actions of an arousal-promoting phase-resetting stimulus.

## Results

### Behavioral Results

#### Effects of Constant Light (LL)

**Figure 1:**
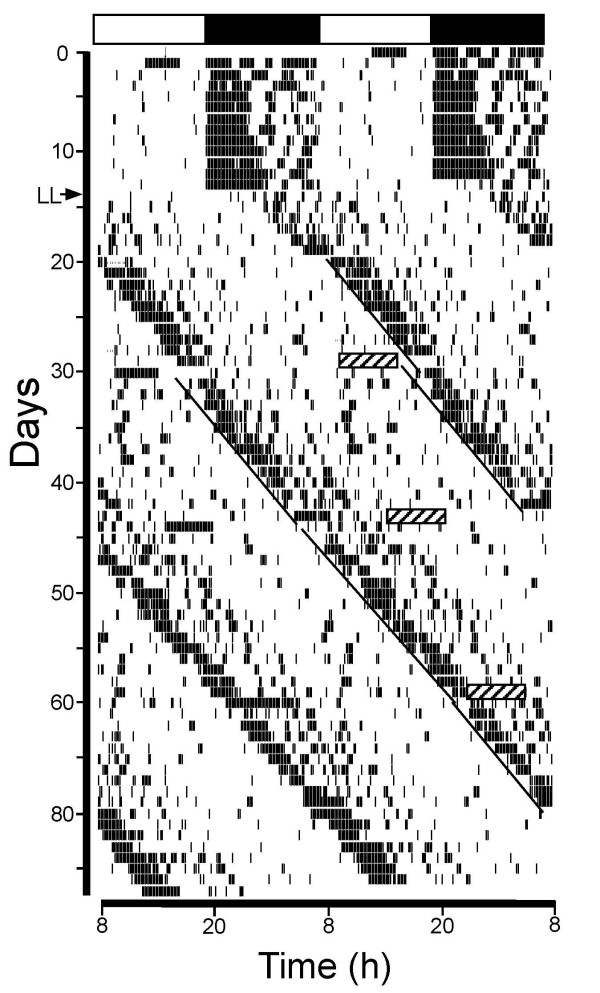
Dark pulses phase-dependently phase-reset murine wheel-running rhythms. Double-plotted wheel-running actogram showing the effects of diurnal (LD) and constant light (LL) lighting regimes and 6 h dark pulses (hatched boxes) on the behavioral activity of a C57BL/6J mouse. Horizontal bar at the top of the actogram depicts the lights-on (unfilled) and lights-off (filled) cycle. Arrow indicates the transfer from LD to LL and black bars indicate locomotion in a running-wheel equipped cage. The solid black lines indicate the onset of the main bout of wheel-running activity (in LL; CT12 convention). The first dark pulse was centered at ~CT6 and elicited a 1.27h phase advance. Subsequent dark pulses delivered at ~CT22 and ~CT13 had negligible phase-shifting effects.

Under LD conditions, all singly housed male C57BL/6J mice (n = 92) showed robust diurnal rhythms in wheel-running activity with most of their locomotor activity occurring during the lights-off phase (Figure 1). With the transition from LD to LL, all mice demonstrated an immediate suppression of wheel-running activity. Under LD conditions the average number of wheel revolutions in the 24 h preceding this transition was 15193 ± 109 (mean ± SEM), and this was significantly reduced by 81% to 2839 ± 43 revolutions during the first 24 h in LL (p < 0.001). With prolonged exposure to LL, the period of wheel-running (tau) significantly lengthened from ~24 h to ~24.8 h (p < 0.001) and the duration of the active phase (alpha) also significantly increased from ~11.8 h to ~13.1 h (p < 0.05) (Table 1). The intensity of wheel-running during alpha was greatly suppressed from ~19.7 in LD to ~5.3 revolutions/min in LL (p < 0.001) and the percentage of variance explained by the dominant period of the running-wheel rhythm (PVE) was also significantly reduced from ~67% in LD to ~27% in LL (p < 0.001), indicating that the strength of the locomotor rhythm diminished in these conditions (Table 1).

**Table 1:** Effects of transition from light-dark to constant light on properties of Murine wheel-running rhythms

**Circadian Parameter**	**Light-Dark (LD)**	**Constant Light (LL)**
tau (h)	24.08 ± 0.03 h	24.84 ± 0.04 h*
Percentage of Variance Explained by the Dominant Rhythmic Component (PV%)	66.6 ± 1.47	26.6 ± 0.98***
Alpha Duration (h)	11.8 ± 0.15	13.1 ± 0.27*
Motor Activity (RPM)	19.7 ± 1.19	5.3 ± 0.51***

***P < 0.001; *P < 0.05

#### Dark Pulse Phase-Response Curve

**Figure 2:**
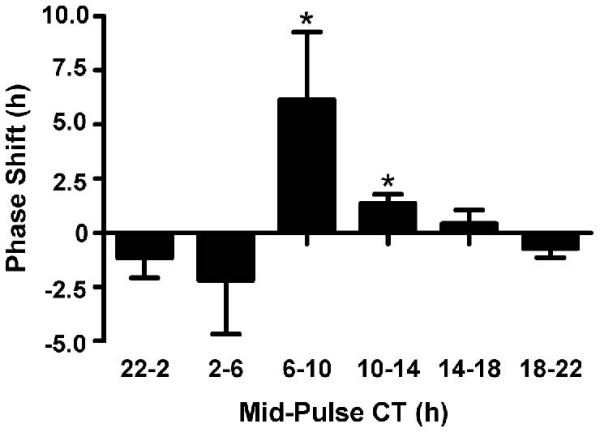
Phase-response curve to 6 h dark pulses of mouse wheel-running rhythms. Histograms represent mean ± 95% CI. Significant advances were elicited by dark pulses centered between CT6-10 and CT10-14, while pulses centered on the late subjective night/early subjective day had inconsistent resetting actions.

Twenty-five mice, from the above 92, received dark pulses of 6 h duration at various points in the circadian cycle. Dark pulses centred between mid-subjective day and early subjective night significantly advanced circadian phase (Figures 1, 2). More specifically, pulses initiated between CT3–7 (n = 16) elicited phase shifts with an average magnitude of ~6.17 h. Dark pulses starting between CT7–11 (n = 9) elicited smaller but still significant phase shifts with an average magnitude of ~1.38 h (mean ± 95% confidence intervals (CI) values did not overlap zero). Dark pulses applied at other circadian times, failed to elicit significant phase shifts (mean ± CI values overlapped zero) (Figure 2). In addition to the resetting actions, dark pulse exposure acutely increased wheel-running by ~2.3 km on the day of the pulse. However, this induced activity did not correlate with the resultant phase shift magnitude (Pearson correlation, p > 0.05; data not shown), suggesting that the dark pulse elicited increase in wheel-running is not a key factor influencing phase shift magnitude to this stimulus. Interestingly, on the days following the dark pulse, the strength of the wheel-running rhythm was significantly reduced (PVE reduced from 31.1% to 28.6%; p < 0.05). Further, although tau and the duration of alpha were not affected by the dark pulse, the intensity of wheel-running during alpha was significantly reduced from ~5.7 to ~4.7 revolutions/min (p < 0.05). Taken together, these data (Table 2) indicate that in addition to phasic resetting, dark pulses have prolonged effects on murine behavioral rhythms.

**Table 2:** Effects of dark pulses on murine wheel-running rhythms

**Circadian Parameter**	**Pre-Dark Pulse**	**Post-Dark Pulse**
tau (h)	24.77 ± 0.05	24.67 ± 0.04
Percentage of Variance Explained by the Dominant Rhythmic Component (PV%)	31.1 ± 1.6	28.6 ± 1.6*
Alpha Duration (h)	12.6 ± 0.5	12.4 ± 0.4
Motor Activity (RPM)	5.71 ± 0.9	4.7 ± 0.73*

*p < 0.05

### Immunohistochemistry

#### Circadian variation in expression and activation of OXA neurons

**Figure 3:**
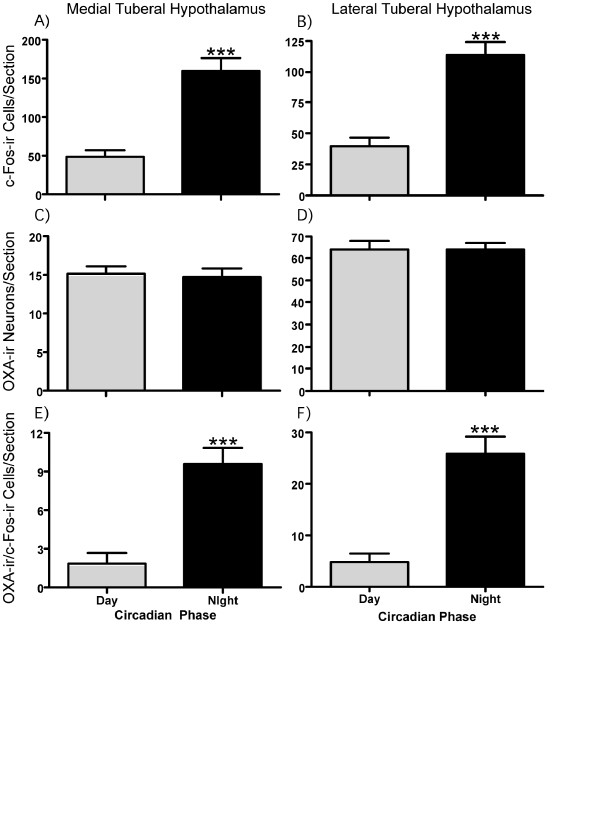
**Circadian day-night profiles of c-Fos and orexin-A expression in the tuberal hypothalamus**. The expression of c-Fos-ir was significantly higher during the subjective night (dark filled histograms) compared with the subjective day (gray filled histograms) in both the medial (**A**) and lateral (**B**) tuberal hypothalamus. Co-expression of c-Fos with OXA neurons showed a similar temporal relationship in medial (**E**) and lateral (**F**) tuberal hypothalamus. The numbers of OXA neurons did not vary from subjective day to night in either the medial (**C**) or lateral (**D**) tuberal hypothalamus. Histograms show mean ± SEM. ***p < 0.001.

Consistent with previous reports in other rodent species [11-13,20,21], OXA-ir was detected within the cytoplasm and processes of a population of hypothalamic neurons. OXA-ir neurons were mostly restricted to the TH, with the lateral TH containing more immunopositive cells than the medial TH. OXA-ir fibres and terminals were found in many brain areas including the PeriSCN region and within the IGL, MRN, and dorsal raphe (DRN) (Additional file 1). Using c-Fos-ir as a marker of neuronal activation, we assessed the numbers of activated cells in the medial and lateral TH as well as within key structures of the neural circadian system. Sections from animals under the LL condition were used as controls against those examined during and after dark pulse exposure. Single-labeled OXA-containing neurons, single-labeled c-Fos-ir, and those OXA-ir neurons double-labeled with c-Fos-ir were counted. In the medial TH, one-way ANOVA indicated a highly significant effect of circadian time on neuronal activation (F_6,23 _= 8.47, p < 0.001). Planned contrast comparison of the mean c-Fos-ir expression level during subjective day (collapsed across CT5, 6, and 9), with the mean c-Fos-ir expression during subjective night (collapsed across CT16, 17, and 20), revealed a highly significant increase in c-Fos-ir during subjective night (p < 0.001; Figure 3A). The number of OXA-ir neurons did not vary as a function of circadian time (p > 0.05; Figure 3C), but there was a highly significant effect of circadian time on the number of OXA-ir cells that co-expressed c-Fos-ir (F_6,23 _= 4.63, p < 0.01); with more OXA-ir neurons activated during the subjective night than the subjective day (p < 0.0001; Figure 3E). A separate comparison of the number of c-Fos-ir OXA neurons at CT9 (1.2 ± 0.5) and CT12 (7.4 ± 2.3) revealed a significant increase (p < 0.05) at the transition to subjective night, indicating that activation of OXA cells in the medial TH begins prior to the onset of vigorous wheel-running. c-Fos-ir in non-OXA neurons did not show this pattern of activation (p > 0.05).

Similar trends were observed in the lateral TH. Here c-Fos-ir expression varied significantly across the circadian cycle (F_6,23 _= 4.44, p < 0.01) with substantially more activated cells visualized from subjective night time points than from the subjective day (p < 0.001; Figure 3B). The number of OXA-ir neurons did not vary across the circadian cycle in LL (p > 0.05; Figure 3D). However, the number of OXA-ir neurons co-expressing c-Fos-ir varied across the circadian cycle (F_6,23 _= 4.91, p < 0.01), with significantly more double-labeled cells detected during the subjective night compared with the subjective day (p < 0.0001; Figure 3F). A separate comparison of the number of c-Fos-ir OXA neurons at CT9 (4.6 ± 2.5) and CT12 (19.7 ± 5.1) revealed a significant increase (p < 0.05) at the transition to subjective night, indicating that activation of OXA cells in the lateral TH begins prior to the onset of vigorous wheel-running. Again, c-Fos-ir in non-OXA neurons did not show this pattern of activation (p > 0.05).

From these data, it is clear that in the lateral and medial TH, cellular activation displays a pronounced circadian profile (including neurons containing OXA). Parametrically, it appears that the lateral TH has more activated OXA-ir neurons at subjective night than in the medial TH. However, when the data are normalized, the medial TH OXA-ir population shows the greater activation, with double-labeled OXA-ir cells rising from ~9.5% during the subjective day to ~60% during the subjective night. In the lateral TH, activated OXA-ir cells rose less dramatically, from ~6.4% of OXA-ir neurons during the subjective day to ~37% during the subjective night. Moreover, OXA neurons in the medial and lateral TH (but not non-OXA neurons) express significantly more c-Fos-ir at CT12. Since detection of c-Fos-ir in neurons typically occurs >60 min following an appropriate stimulus [22], these increases cannot be attributable to the commencement of wheel-running at CT12 and instead must be due to a circadian signal. Activity in the SCN of nocturnal mice is high during the behaviorally quiescent subjective day and low during the active subjective night. Available data indicate that SCN output signals suppress locomotor activity during the day, with the decline of this output at late day/early subjective night permitting locomotor activity. Therefore, the maximal levels of c-Fos/OXA co-expression seen around the mid-subjective night are presumably due to the combined actions of circadian disinhibition and feedback from behavioral arousal including forward locomotion.

#### Do dark pulses activate orexin neurons?

**Figure 4:**
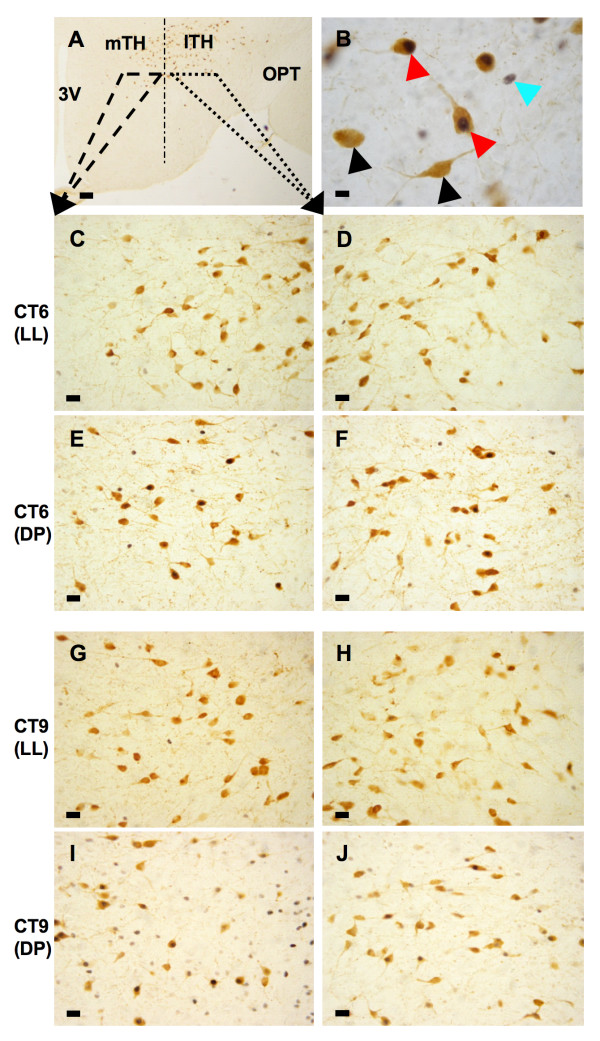
**Mid-subjective day dark pulses activate orexin-A neurons in the tuberal hypothalamus**. Photomicrographs illustrating c-Fos-ir and OXA-ir cells as well as c-Fos/OXA double-labeled cells within the TH. Panel (**A**) shows a low magnification representation of the regions shown in subsequent panels. Panel (**B**) shows OXA-ir cells (black arrows), a c-Fos-ir nucleus (blue arrow) and c-Fos/OXA double-labeled cells (red arrows) within the TH at high magnification. 3 V = third ventricle; lTH = lateral tuberal hypothalamus; mTH = medial tuberal hypothalamus; OPT = optic tract. Calibration bars = 100 μm (**A**), 10 μm (**B**), 25 μm (**C-J**).

We examined whether a stimulus, which promotes arousal and phase-dependent SCN resetting, could alter cellular activation within the medial and/or lateral TH. This was established by administering a 6 h dark pulse, initiated at either CT5 or 16, and determining the expression of c-Fos-ir in both OXA-ir positive and negative cells. At these timepoints, the SCN is most and least responsive respectively, to the phase-resetting actions of a dark pulse (see dark pulse PRC above). Mice dark pulsed at CT5 were sampled 1 h (CT6) and 4 h (CT9) following pulse onset, and 1 h after reinstatement of light (CT12). Animals dark pulsed at CT16 were sampled 1 h (CT17) and 4 h (CT20) following the onset of dark. All sets of tissue were compared to time-matched unpulsed LL controls. Representative photomicrographs showing c-Fos-ir and OXA-ir the medial and lateral TH at CT6 and CT9 under unpulsed and pulsed conditions are shown in Figure 4. In all samples, the number of detectable OXA-ir cells did not change significantly between animals or lighting condition (data not shown).

**Figure 5:**
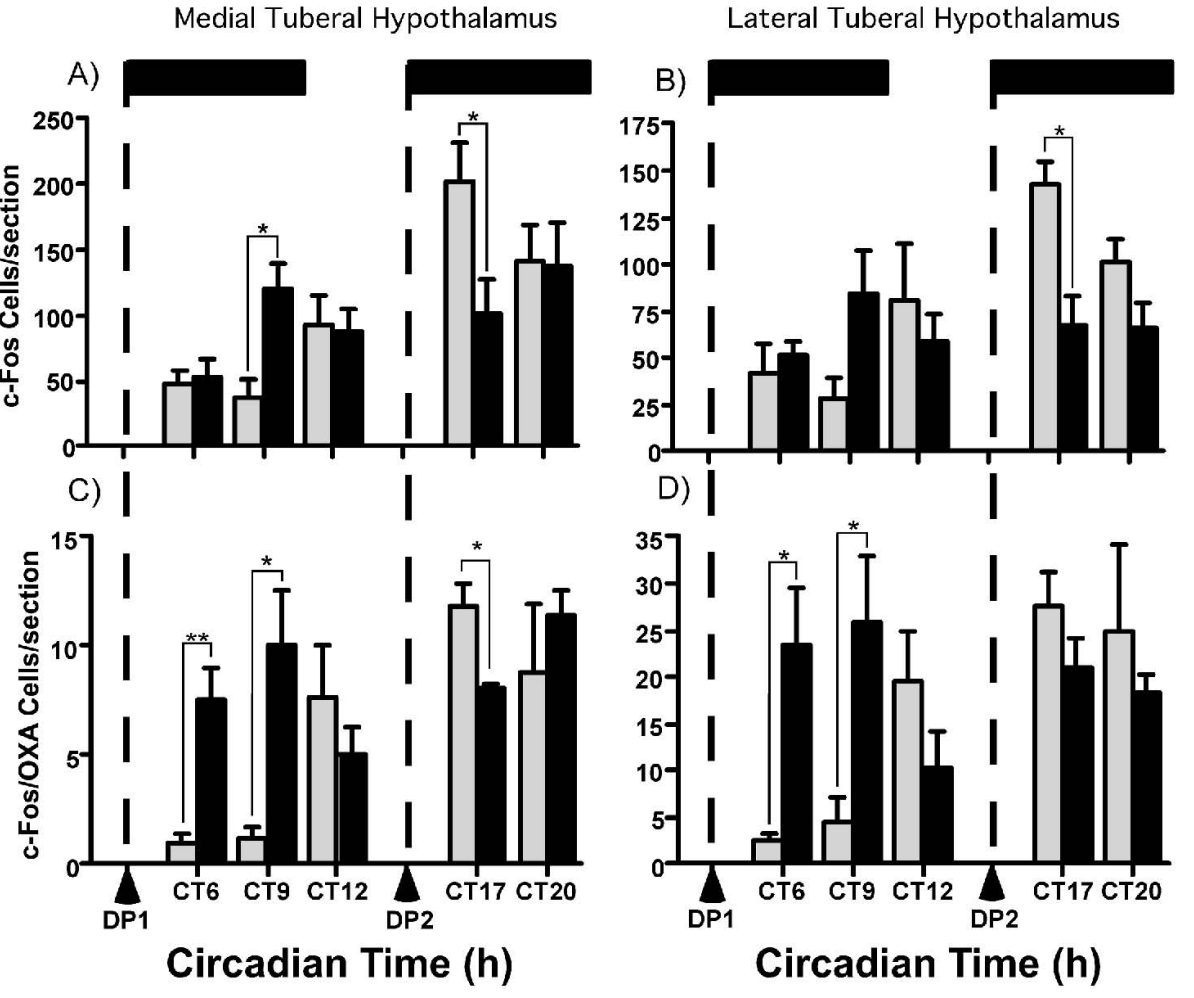
**Circadian and dark pulse regulation of orexin-A and non-orexin-A neuronal activation in the tuberal hypothalamus**. c-Fos expression (**A**) and c-Fos co-expression in OXA neurons (**C**) are increased in the medial TH by a dark pulse (DP1; onset indicated by the left vertical broken line at DP1 arrowhead, while the 6 h duration of DP1 is shown as the filled horizontal bar top left) beginning at CT5, while after 1 h exposure to a 6 h dark pulse (DP2; beginning at the right broken vertical line and arrowhead at CT16 with the 6 h duration, depicted by the filed horizontal bar top right) suppresses c-Fos and c-Fos/OXA co-expression. In the lateral TH c-Fos expression is not altered by the subjective day dark pulse (**B**), but co-expression of c-Fos with OXA neurons is elevated 1 h and 4 h into the pulse (**D**). By contrast, c-Fos expression in the subjective night in the lateral TH is suppressed within 2 h (**B**); co-expression of c-Fos with OXA was unaffected. Histograms show mean ± SEM. **p < 0.01, *p < 0.05.

In the medial TH, dark pulse exposure at CT5, significantly elevated c-Fos-ir 4 h (CT9) into the pulse (p < 0.05; Figure 5A), but not at other timepoints (CT6 and CT12) examined. In the lateral TH, dark pulse exposure had no significant effects on c-Fos-ir at these timepoints (Figure 5B). In comparison, in medial and lateral TH, the number of OXA-ir neurons expressing c-Fos increased significantly at both the CT6 and CT9 analysis time-points (medial TH: p < 0.01 and p < 0.05 respectively and lateral TH: both p < 0.05 respectively; Figures. 4C–J and 5C, D). Further, the extent of co-localization differed modestly between areas. More than half the total medial OXA-ir cells (~58%) became activated during the pulse, whereas ~40% of lateral TH OXA-ir cells were activated. Additionally, the finding that non-OXA-ir cells in medial TH become activated by this stimulus, establishes that dark pulse activation is a feature exhibited by many cells types in this part of the hypothalamus.

For animals that were exposed to a dark pulse beginning at CT16, no significant change in total c-Fos-ir was detected in the medial TH at any time-point sampled. However, there was a significant suppression of OXA-ir activated cells at 1 h (CT17) after pulse initiation (p < 0.01; Figure 5C) with the proportion of activated OXA-ir cells reducing from ~60% to ~52%. Conversely, lateral TH expression of c-Fos-ir was significantly reduced 1 h (CT17) but not 4 h (CT20) into the pulse (p < 0.05; Figure 5B). There was no effect on activated OXA cells at either CT (Figure 5D). Overall, a dark pulse centered around the end of the subjective night modestly, and transiently, suppressed OXA activation in the medial TH, while reducing non-OXA cell activation levels in the lateral TH.

#### Effect of a dark pulse on c-FOS-ir within the SCN, IGL, MRN and DRN structures

**Figure 6:**
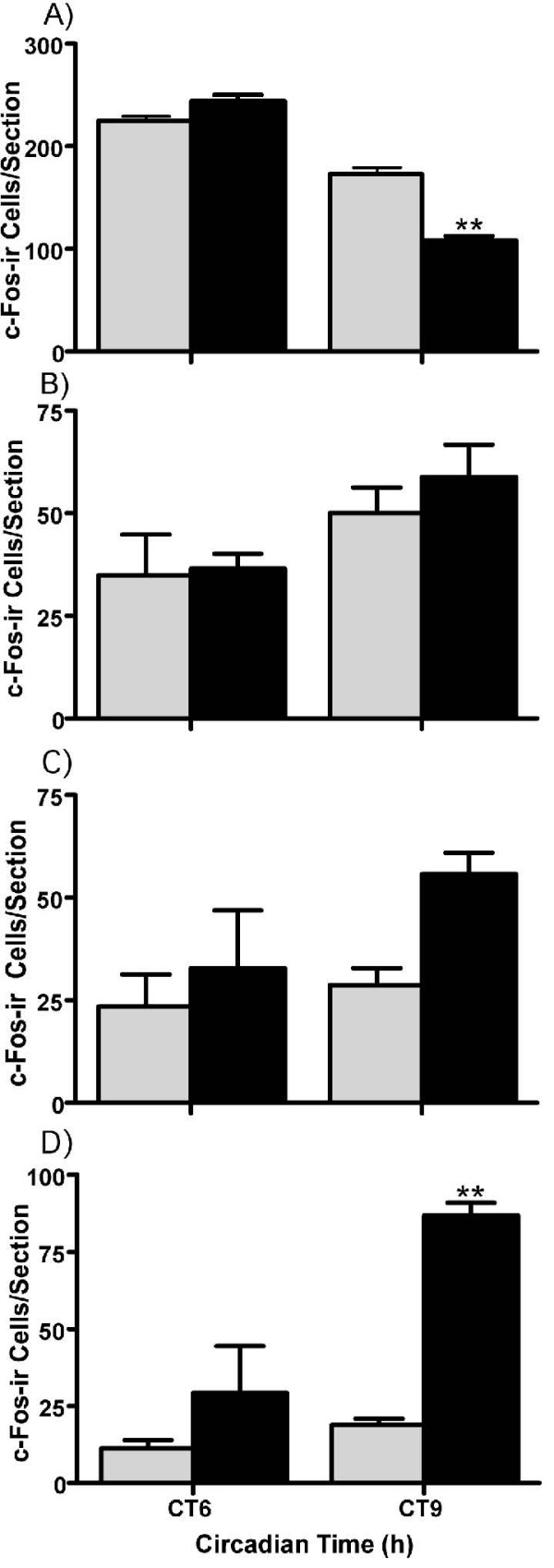
**Mid-subjective day dark pulses suppress the suprachiasmatic nuclei and activate the median raphe**. Histograms show expression of c-Fos (mean ± SEM) at CT6 and CT9 in constant light (gray-filled histograms) and 1 h and 4 h into a 6 h dark pulse beginning at CT5 (black-filled histograms) in the SCN (**A**), IGL (**B**), DRN (**C**), and MRN (**D**). **p < 0.01.

Since OXA-ir neurons of the TH are anatomically linked to many components of the neural circadian system, including the SCN, IGL, MRN, and DRN, we assessed if the activational state of cells in these regions varied in response to a dark pulse beginning at CT5. In unpulsed LL controls at CT6 and CT9, c-Fos-ir was spontaneously expressed throughout all levels of the SCN. At 4 h, but not 1 h, into the 6 h dark pulse, c-Fos-ir in the SCN was significantly reduced (unpaired t-test, p < 0.01 Figure 6A). Qualitatively, the area most prominently suppressed by the dark pulse appeared to be the rostral SCN and preliminary inspections indicated both 'core' and 'shell' regions [23] of the mid-rostrocaudal SCN were similarly affected by the dark pulse (data not shown). In both the IGL and DRN, small but non-significant increases in c-Fos-ir were coincident with dark pulse exposure (Figure 6B, C). More robust changes in cellular activation were observed within the MRN, where the number of c-Fos-ir cells became significantly elevated 4 h into the dark pulse (unpaired t-test, p < 0.01; Figure 6D). Collectively, these data indicate that a dark pulse given during the subjective day significantly and differentially affects cell activation in the neural circadian system, specifically suppressing SCN cells while activating MRN cells.

## Discussion

This study demonstrates unequivocally that orexin neuronal activation is under pronounced circadian control and further that orexin neurons are selectively activated by an SCN-resetting arousal-promoting stimulus. We also highlight potential differences in the activation state of orexin neurons in the medial and lateral TH. These findings reveal new complexities of SCN-TH interactions in the regulation of brain states and behavior.

Consistent with studies in Syrian hamster and Swiss mouse, we found that a 6 h dark pulse robustly phase-advances C57BL/6J wheel-running rhythms when initiated during the mid- to late subjective day [24-30]. Further, our observation that the magnitude of dark pulse-evoked wheel-running activity does not necessarily correlate with the magnitude of the phase shift is also in broad agreement with other studies [31-33]. Previously, dark pulses given during the late subjective night/early subjective day were found to elicit phase-delays in Syrian hamster and Swiss mouse wheel-running rhythms [24-26]. Although we did observe that some C57BL/6J mice phase-delayed to dark pulses given at this time of the circadian cycle (see Figures 2 and 3), delays were not consistently evoked. This discrepancy is likely to be due to a *bona fide *species-related difference, as we found that Syrian hamsters housed under identical conditions invariably show significant phase-delays to 6 h dark pulses beginning around CT16 and extending to early subjective day [26]. Collectively, these data suggest that the C57BL/6J mouse is more readily phase-advanced than phase-delayed by 6 h dark pulses.

Our study complements and extends earlier research in which temporal variation in orexin cell activation was determined primarily under diurnal conditions [20,34,35] or limited (two time points) circadian sampling frequencies [17]. Under LL conditions in which the amplitude and intensity of murine wheel-running are greatly suppressed, we detected a highly significant circadian profile in the levels of activated of OXA-ir and non-OXA-ir neurons in the medial and lateral TH. Here, neurons demonstrated substantially higher levels of c-Fos-ir during the subjective night phase. Interestingly, we detected significant (but sub-maximal) increases in OXA-ir/c-Fos-ir double-labeled neurons in both the medial and lateral TH at CT12 compared to CT9. Because detection of c-Fos-ir in neurons typically occurs ~60 min following an appropriate stimulus, this is evidence that OXA cells are not simply reactive to the initiation of locomotor activity at CT12 and are subject to genuine circadian control. However, because maximal levels of OXA activation are not seen until the mid subjective night (when locomotor activity is well established) (see Figure 5C, D) it is likely that behavioral feedback mechanisms further activate OXA cells. This circadian pattern of orexin neuron activation is consistent with the circadian profile of orexin detected in the cerebrospinal fluid of nocturnal rodents; the amplitude of which is suppressed by exposure to LL and abolished in SCN-lesioned animals [36,37]. This indicates that c-Fos-labelling as a marker of neuronal activation corresponds with the release of OXA.

The effects of dark exposure during the mid-subjective day on c-Fos-ir expression in the circadian system support earlier findings from Syrian hamster [27]. A significant suppression of c-Fos-ir in the SCN was detected during the daytime dark pulse. This was accompanied by activation of MRN neurons, OXA-ir and non-OXA-ir neurons in the medial TH, and OXA-ir neurons in the lateral TH. Projections from cells in the IGL and MRN to the SCN are both important for the phase-advancing actions of various non-photic stimuli [3,8,38]. However, we observed that unlike the MRN, daytime dark pulses did not robustly activate neurons in the IGL and DRN. This suggests that cells in MRN are more involved in dark pulse evoked phase shifts than those of the IGL or DRN. Consistent with this interpretation, Coogan and Piggins [27] did not observe significant increases in c-Fos-ir labeling in the hamster IGL following a daytime dark pulse. Similarly, Harrington and Rusak [28] found that IGL lesions do not abolish the resetting actions of dark pulses in Syrian hamsters. Although other non-photic stimuli, such as running in a novel wheel, can induce c-Fos-ir in the hamster IGL [39,40], activation of the geniculo-hypothalamic tract (GHT) does not appear to be critical for dark-pulse evoked phase advances. Electrical stimulation of GABA and serotonin containing-neurons in the MRN reduces light-evoked c-Fos-ir in the SCN and similar effects have been documented with exogenous application of serotonergic or GABAergic agonists into the SCN [8,41-43]. Moreover, analogous to daytime dark pulses, serotonergic agonists given during the subjective day phase advance rodent wheel-running rhythms and suppress clock gene expression in the rodent SCN [44,45]. Further, serotonergic agonists given during the subjective day can potentiate dark pulse evoked phase advances, supporting the contention that the MRN-SCN pathway is involved dark pulse resetting of circadian rhythms [45].

Within the TH, our results suggest functional differences in the circadian and photic regulation of both orexin and non-orexin neuronal groups. Cells in the TH demonstrated circadian variation in c-Fos-ir expression, and in the degree of activated OXA-ir neurons. In the medial and lateral TH, the basal level of OXA-ir activation during the subjective day is similar (~10% and ~7% respectively), but across subjective night, the medial TH OXA-ir cells show a larger activation, with ~60% containing c-Fos-ir while ~42% of lateral OXA are activated. When exposed to a 6 h dark pulse during the subjective day, it is also the medial TH that expresses the largest increase in the proportion of OXA-ir neurons co-expressing c-Fos-ir. Complementary findings have been published recently by Webb et al. [40] who report that hamsters confined to running wheels (an arousal-inducing procedure) demonstrate a greater proportion of OXA-ir/c-Fos-ir double-labeled cells in medial TH areas compared with lateral TH areas.

Intriguingly, dark pulse exposure at subjective night causes a small but significant decrease in OXA-ir/c-Fos-ir co-localization in the medial TH, without affecting the activational state of non-OXA-ir cells. A different pattern is seen in the lateral TH; here it is the non-OXA-ir cells whose activation is suppressed, while the OXA-ir neurons are not significantly affected. These data support suggestions that different populations of orexin neurons undergo differential tonic regulation and respond differently to various stimuli [17,46,47]. Here, activation of OXA neurons in the medial TH appears more tightly linked to circadian regulation of behavioral arousal than those located laterally. This may be a reflection of the extensive innervation of medial hypothalamic sites by SCN efferents and/or their proximity to the third ventricle, in which cerebrospinal fluid borne SCN-derived signals can be communicated [48]. Additionally, SCN-controlled peripheral signals such as corticosterone can potentially alter orexin cellular activity [49].

**Figure 7:**
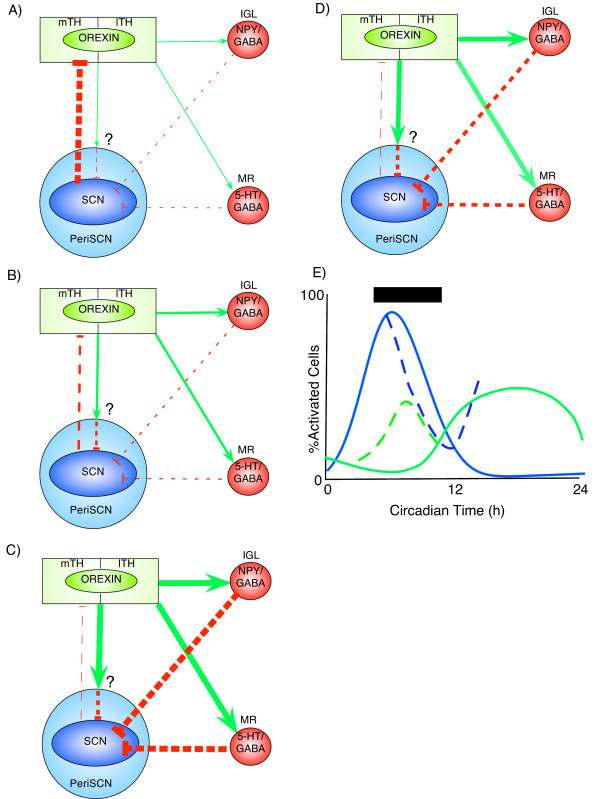
**Circadian and dark pulse regulation of orexin cellular activation**. (**A**) Mid-subjective day: inhibitory output (broken red lines) from the SCN is high, with cells in the medial (mTH) and lateral tuberal hypothalamus (lTH), including orexin neurons, minimally active with little/no excitatory outputs (green arrows) to the IGL and median raphe (MR) whose cellular activity/output is also low. Orexin efferents may (**?**) act via the PeriSCN to suppress SCN activity [53]. (**B**) Late subjective day: SCN inhibitory output reduced, orexin cells submaximally activated, with increased excitatory output to the PeriSCN, IGL, and MR. (**C**) Mid-subjective night: SCN inhibitory output minimal, orexin cellular activation maximal. Increased excitatory input to the PeriSCN, IGL and MR increases inhibitory outputs to the SCN. (**D**) Dark pulse at mid-subjective day interrupts retinal input, reducing SCN activity and its inhibition of the mTH and lTH. Disinhibited orexin cells excite PeriSCN, IGL and MR neurons whose outputs suppress the SCN. (**E**) Circadian variation in SCN (blue line) and orexin (green line) activation and the effects of a daytime dark pulse (timing and duration indicated by filled horizontal box) on this activation (broken lines). In (**A-D**), the width of the lines indicates the level of excitatory or inhibitory signal. NPY = neuropeptide Y; 5-HT = serotonin; GABA = γ-aminobutyric acid.

Orexin neurons are ideally positioned to integrate arousal and circadian cues. The results of the present study, as well as those of other researchers, indicates that the pronounced difference in the activation state of orexin neurons from the quiescent subjective day to the behaviorally active night, is likely caused by both circadian influences and arousal/behavioral feedback. During the subjective day, SCN neuronal activity is maximal and, as the SCN output signal is believed to be predominantly inhibitory, acts to suppress other brain areas including the TH (Figure 7A, E). When neuronal activity in the SCN declines, as seen before subjective night, there is a significant rise in c-fos expression in orexin cells which occurs at least 1 h prior to the onset of vigorous subjective nighttime wheel-running (Figure 7B, E). Together, the alteration in SCN input results in a disinhibition of orexin neurons leading to arousal and priming of the animals behavior for waking. Further sustained activation of orexin neurons during the subjective night arises through behavioral feedback and activation of IGL-SCN and MRN-SCN pathways, which in turn, facilitate sustained suppression of the SCN (Figure 7C, E). Accordingly, the cessation of subjective night develops through increased SCN neuronal output through the late night and early subjective day.

Phase-shifting actions of dark pulses can also be explained by similar mechanisms. Application of a daytime dark pulse causes an interruption of the excitatory photic input to the SCN, which results in reduced inhibitory output drive from the SCN. The consequence of this is disinhibition and activation of the orexin cells, and other neuronal populations, to promote arousal and behavioral feedback (Figure 7D, E). The latter effect activates the MRN-SCN pathway, leading to further suppression of SCN neurons. Termination of the dark pulse restores the excitatory light input to the SCN, activating SCN cells and elevating inhibitory output signals to many brain regions, ending neuronal activation in orexin and non-orexin cells. Based on our present data, we predict that MRN-SCN projections are more important than IGL-SCN projections under these conditions. This is in contrast to other arousal promoting non-photic stimuli, such as novelty induced locomotion, for which the GHT projection between the IGL and the SCN appears critical for clock resetting [32].

An additional contributing factor to the phase resetting actions of daytime dark pulses is sleep deprivation. In Syrian hamsters, it is reported that it is sleep deprivation occurring during the dark pulse rather than wheel-running activity that determines phase-resetting to this stimulus [33]. Here, in our study on mice, wheel-running activity during the pulse does not correlate with the magnitude of the phase shift. However, in a small cohort of mice (n = 4) visually monitored with infra-red goggles during the dark pulse given CT5–11, we found that the mice rested a lot less and engaged in many more active behaviors (general exploration, cage-top climbing, etc) than had been recorded in these same animals over CT5–11 in LL conditions (Marston and Piggins, unpublished observations). Since orexin release is enhanced by sleep deprivation [50], this indicates another mechanism via which dark pulses can regulate these and other TH cells.

OXA may also have other direct and indirect effects on SCN neuronal activity. For example, in addition to innervating the IGL and MRN, OXA-ir fibres project to the Peri-SCN region [12,51] and OX1R protein is detectable within the rat SCN [52]. This raises the possibility that locally released OXA may act on the SCN. Indeed, we recently showed that OXA and OXB can alter rat SCN neuronal activity in vitro, with some actions involving recruitment of GABAergic mechanisms [53]. Such actions of locally released orexins on the SCN may feedback to modulate TH cellular activity since one study has described SCN projections to OXA neurons [15].

## Conclusion

Collectively, our findings raise the possibility that activation of OXA neurons is involved in resetting of the SCN circadian clock and further allude to the pathway from the MRN to the SCN being functionally more important than the GHT for dark pulse-induced phase-shifting. Moreover, our findings identify at least two separate populations of OXA neurons that are differentially activated by endogenous and exogenous cues, thereby enabling the orexin system to collectively 'multitask' and coordinate appropriate physiological and behavioral states.

## Methods

Adult male C57BL/6J mice were purchased from Harlan (Loughborough, UK). All procedures were carried out in accordance the Animal (Scientific Procedures) act, 1986 (UK). Animals were initially group housed (6–8 animals per cage) for 1–2 weeks under a 12 h:12 h light/dark cycle (LD; lights-on 07:00) before being individually housed in running wheel-equipped (stainless steel, 16 cm diameter, North Kent Plastics, Kent, UK) polycarbon cages (overall dimensions 45 cm × 21 cm × 19 cm). Individually housed mice were kept under 12 h:12 h LD cycle for a further 10 days and then released into constant light (LL; light intensity at cage level ~100 lux) to establish stable free-running activity rhythms for at least 10 circadian cycles prior to experimental procedures. Food (B&K Universal, Hull, UK) and water were available *ad libitum *in all conditions.

Each revolution of the running wheel depressed an externally mounted microswitch, the activation of which was recorded and counted via a PC using the collect module of the Chronobiology Kit software suite (Stanford Software Systems, Santa Cruz, CA, USA). Actograms of wheel-running rhythms and wheel revolution counts were produced with the Analyze 9 module (Stanford Software Systems). Changes in endogenous period (tau) under LD and LL conditions were estimated by the chi-square periodogram. Wheel-running activity rhythms were visualized offline as double-plotted actograms. The El Temps programme (Dr. Noguerra, University of Barcelona, Barcelona, Spain) was used to measure a number of key properties of the wheel-running rhythm and to determine the effects of exposure to LL and dark pulses (see [26] for further details).

### Dark Pulse Phase-Response Curve

To generate a phase-response curve (PRC) to dark pulses, mice (n = 25) were given a 6 h dark pulse every 10–16 days at various points across the circadian cycle. Dark pulses were initiated by turning off the room lights for 6 h and terminated by reinstatement of light. Animals remained undisturbed in their home cages throughout the dark pulse. All animals received at least one dark pulse, with some animals receiving up to 3 pulses over 60 circadian cycles.

Steady state phase shifts were independently assessed by three experienced researchers (two blind to experimental conditions) using the line of best-fit method [25,27,54]. Briefly, two lines of best fit were drawn through the onsets of wheel-running activity (CT12 by convention). The first line delineated activity onset (CT12) over the 7 cycles prior to the dark pulse. This line was extrapolated to predict CT12 on the first cycle following the pulse. The second line identified activity onset over the 7–10 cycles following the dark pulse (for the purposes of line-fitting, circadian cycles containing transients were discounted from this process). Interpolation of the second line also allowed CT12 to be predicted on the first cycle following the dark pulse. The difference (in min) between the CT12 values obtained by extrapolation and interpolation was taken to be the steady state phase shift elicited by the dark pulse. Animals with unstable behavioral rhythms were excluded from analysis.

Steady state phase shifts in response to dark pulses across the circadian cycle were initially graphed on a scatterplot to aid visualization. For analysis, phase shift data were grouped into 4 h bins (e.g. dark pulses centered between CT2–6, 6–10 etc). Each bin contained at least 6 data points (range n = 6–16) with no animal contributing more than a single data point to any individual CT bin. Where an animal received more than a single pulse within a specific 4 h period the values were averaged to provide a single value. The relationship between intensity of wheel-running activity induced by a subjective day dark pulse and the magnitude of resulting phase shifts was examined by parametric correlation.

### Immunohistochemistry

In the immunolabeling experiment, mice (n = 67) were sampled either in LL (for use as time-matched unpulsed controls), or at 1 h or 4 h into a 6 h dark pulse (initiated at either CT5 or CT16). In addition, some mice were sampled 1 h after cessation of the CT5 dark pulse (CT12). Animals were culled by cervical dislocation and decapitation under halothane anesthesia. Brains were carefully removed from the skull and placed in fixative [4% paraformaldehyde in 0.1 M phosphate buffer (PB)] for 4 days at 4°C. Following cryoprotection (30% sucrose in 0.1 M PB solution), brains were rapidly frozen with crushed dry ice and mounted onto the cryostage of a sledge microtome (Series 8000, Bright Instruments Ltd., Huntingdon, UK) and cut into 30 μm thick coronal sections (four serial sets of sections per brain).

Free-floating sections were washed 3 × 10 min in 0.1 M PB then incubated for 20 min in 1.5% hydrogen peroxide made in 0.1 M PB (Sigma, Poole, UK) to remove endogenous peroxidase activity. Sections were washed (3 × 10 min in 0.1 M PB) and subsequently incubated for 60 min in a blocking solution of 5% normal donkey serum (NDS; Sigma, Poole, UK) in 0.1 M PB; then transferred into the primary antibody (made up in 5% NDS) for 36–48 h at 4°C. The primary antibodies used were polyclonal goat anti-orexin-A (1:1000; Santa Cruz Biotechnology, Santa Cruz, CA, USA) and polyclonal rabbit anti-c-Fos (1:8000; Santa Cruz Biotechnology).

After incubation the sections were returned to room temperature (22°C) and washed in 0.1 M PB (3 × 10 min) before addition for 90 min in an appropriate biotinylated IgG secondary antibody (1:400 donkey anti-goat or 1:400 donkey anti-rabbit in 0.1 M PB; Jackson Immunoresearch, West Grove, PA, USA). Sections were washed (3 × 10 min in 0.1 M PB) then transferred to avidin-biotin complex (ABC) solution (1:200; Vector Laboratories, Peterborough, UK) for 90 min. Slices were then prepared for enzymatic detection after two further 10 min 0.1 M PB washes. For c-Fos-ir development, sections were placed for 10 min in a 0.1 M sodium acetate solution (pH 6.0) and then exposed to a nickel-intensified diaminobenzidine chromagen (NiDAB). The reaction was catalyzed with 0.015% glucose oxidase (Sigma), yielding a blue/black reaction stain. For OXA-ir double-labeling, sections were returned to 1.5% hydrogen peroxide before being rerun in the previous steps with the corresponding primary and secondary antibody until enzymatic detection. Diaminobenzidine chromagen without nickel intensification (DAB), catalyzed with 0.015% hydrogen peroxide produced a red/brown reaction product indicating OXA detection. The reaction was quenched in dH_2_O. Stained sections were rinsed in ~0.005 M PB and mounted onto gelatin coated slides and air-dried. They were then dehydrated through a series of graded ethanol baths (70%, 95% and 2 × 100%), cleared in Histoclear (National Diagnostics, Hull, UK) and air-dried before coverslipping.

Sections were visually inspected under an Olympus BX-50 microscope and photographed with an attached digital camera (Olympus c-4000 z). High resolution digital images of representative rostral, intermediate and caudal SCN, IGL, MRN and DRN sections were taken and imported into Microsoft PowerPoint (XP edition; Microsoft, Seattle, WA, USA). The SCN, IGL, MRN and DRN were delineated from adjacent neuroanatomical structures with reference to a stereotaxic atlas [55] and a series of cresyl violet stained C57BL/6J brain sections (provided by Dr David Cutler). Another experienced researcher confirmed accuracy of neuroanatomical delineation. OXA-ir neurons and c-Fos-ir nuclei were counted directly under microscope examination. Two sections (both hemispheres) per rostral, mid- and caudal level of the TH were analyzed from each animal (6 sections/animal). Using a modification of the medial/lateral subdivisions described by other researchers [17,46], we divided the TH into two major divisions, medial and lateral, defined by two counting boxes (each 1.05 mm in height × 0.7 mm in width) corresponding to stereotaxic coordinates from [55] with the medial box: ~4.7–~5.75 mm height × ~0.7 mm from midline; and the lateral box: ~4.7–~5.75 mm height × ~0.7–~1.4 mm from midline) single-labeled and co-localized cells were counted (See additional file 2). All counts were conducted blind to experimental conditions and verified by an independent researcher experienced in cellular and nuclear counting techniques. Immunostaining is expressed as average values/animal/timepoint.

### Statistical analysis

To evaluate the effects of constant light on key features of the wheel-running rhythms we used paired t-tests. To determine the significance of phase shifts to dark pulses, we collapsed the data into 4 h circadian time bins and compared the mean ± 95% confidence interval with 0 min shift and interpreted the absence of an overlap as significant. One way analysis of variance was used for evaluating the significance of circadian time on c-Fos and OXA cellular labeling, with planned comparisons of mean levels at day-night phases. Unpaired t-tests were used as described. The JMP version 6 (SAS Institute, Cary, NC) and Prism Graphpad version 4 (Graphpad Software Inc. San Diego, CA) software packages were used for statistics and construction of graphs.

## Abbreviations

DRN: Dorsal raphe; GHT: geniculohypothalamic tract; IGL: intergeniculate leaflet; LD: light-dark; LL: constant light; MRN: median raphe; OXA: orexin A; PRC: phase-response curve; PVE: percentage of variance; SCN: suprachiasmatic nuclei; TH: tuberal hypothalamus.

## Competing interests

The authors declare that they have no competing interests.

## Authors' contributions

This manuscript is based on doctoral research completed by OJM with additional analysis and interpretation by RHW. OJM, RHW, and RES did the immunohistochemistry and quantification of immunostaining, OJM and MMC analyzed the behavioral data, and NU advised and provided financial support for the study, while OJM and HDP performed the statistical tests. OJM, RHW, and HDP wrote the paper.

## Supplementary Material

Additional file 1 Orexin-A-ir in the mouse hypothalamus, thalamus, and brainstem. Photomicrographs demonstrating regions with moderate to dense populations of orexin-A-ir neurons in the tuberal hypothalamus (arranged rostral to caudal, **A **to **D**) and orexin-A-ir fibres in structures of the circadian system including the suprachiasmatic nuclei (**E**), intergeniculate leaflet (**F**), dorsal raphe (**G**), and median raphe (**H**). 3 V = third ventricle; AHA = anterior hypothalamic area; DMH = dorsomedial hypothalamic area; DR = dorsal raphe nucleus; dLGN = dorsal lateral geniculate nucleus; f = fornix; IGL = intergeniculate leaflet; LHA = lateral hypothalamic area; mlf = medial longitudinal fasciculus; MPA = medial preoptic area; MR = median raphe nucleus; OX = optic tract; PMR = pontine median raphe nucleus; SCN = suprachiasmatic nucleus; TC = tuber cinereum; ts = tectospinal tract; vLGN = ventral lateral geniculate nucleus. Calibration bars = 100 μm (**A-D**), 50 μm (**E, G, H**), 25 μm (**F**). Click here for file

Additional file 2 Depicting the 'counting' boxes used to quantify c-Fos-ir and orexin-A-ir cells in the medial and lateral TH. Depiction of 'counting boxes' used to delineate the medial and lateral tuberal hypothalamus regions (at this level of the rostrocaudal axis) in which neurons single and double-labeled for c-Fos and orexin-A immunoreactivity were quantified. Image taken from Franklin and Paxinos (2001). VMH = ventromedial hypothalamus; 3 V = third ventricle; Arc = arcuate nucleus. Click here for file
