# Supplementary figures and images for: Circadian and dark-pulse activation of orexin/hypocretin neurons
Source: Mol Brain. 2008 Dec 3;1:19. doi: 10.1186/1756-6606-1-19 (PMC2632999; doi:10.1186/1756-6606-1-19)

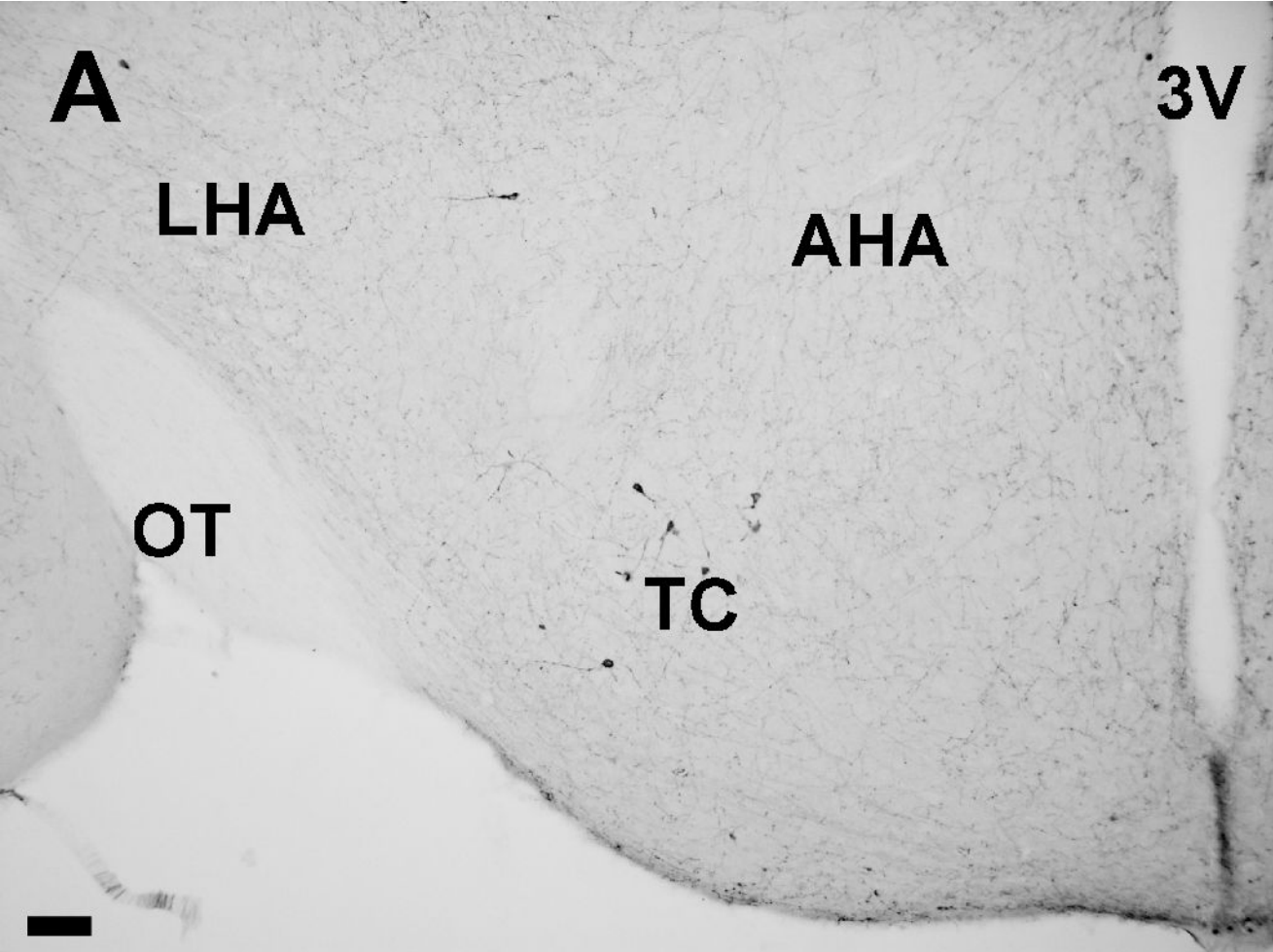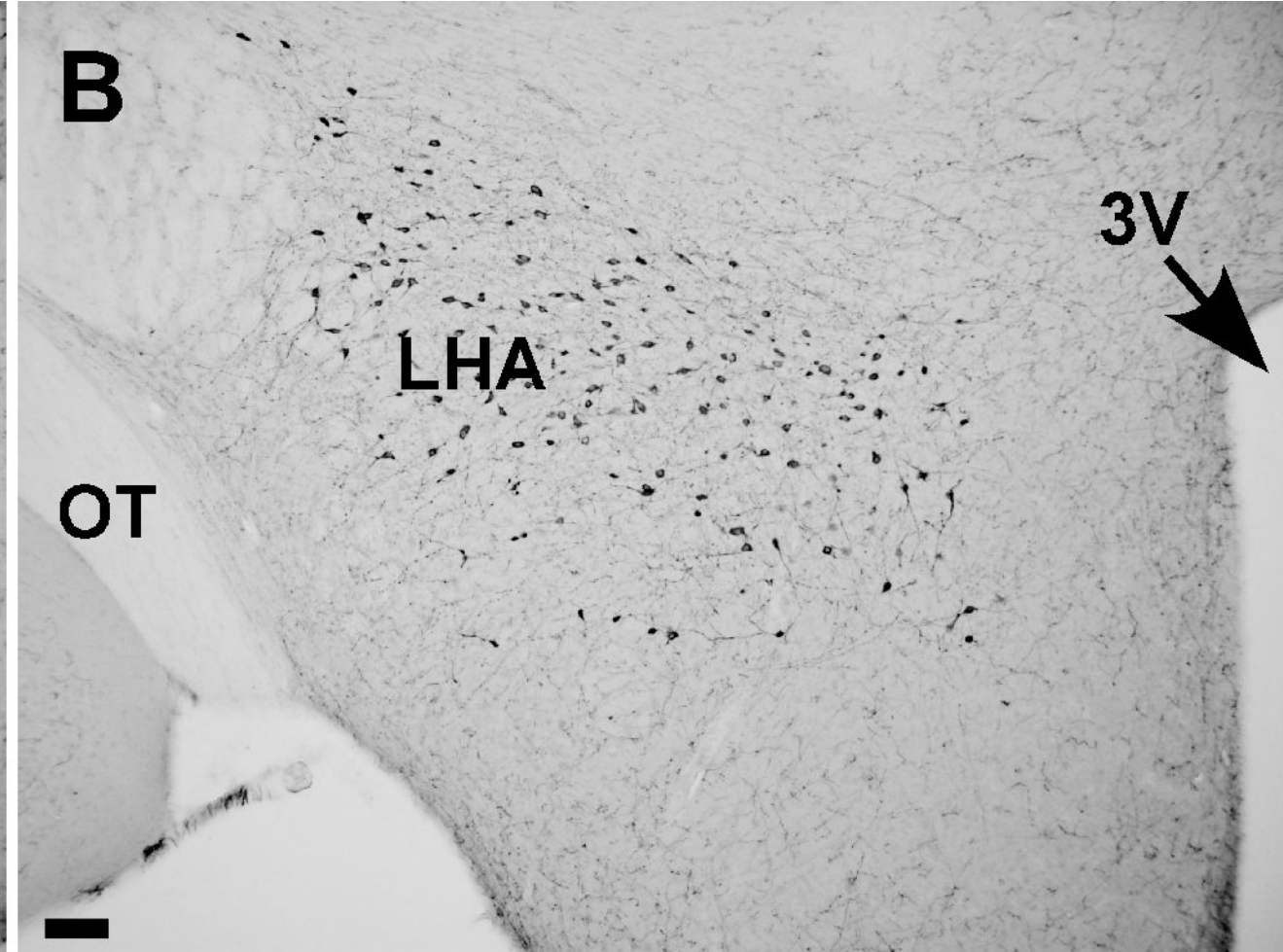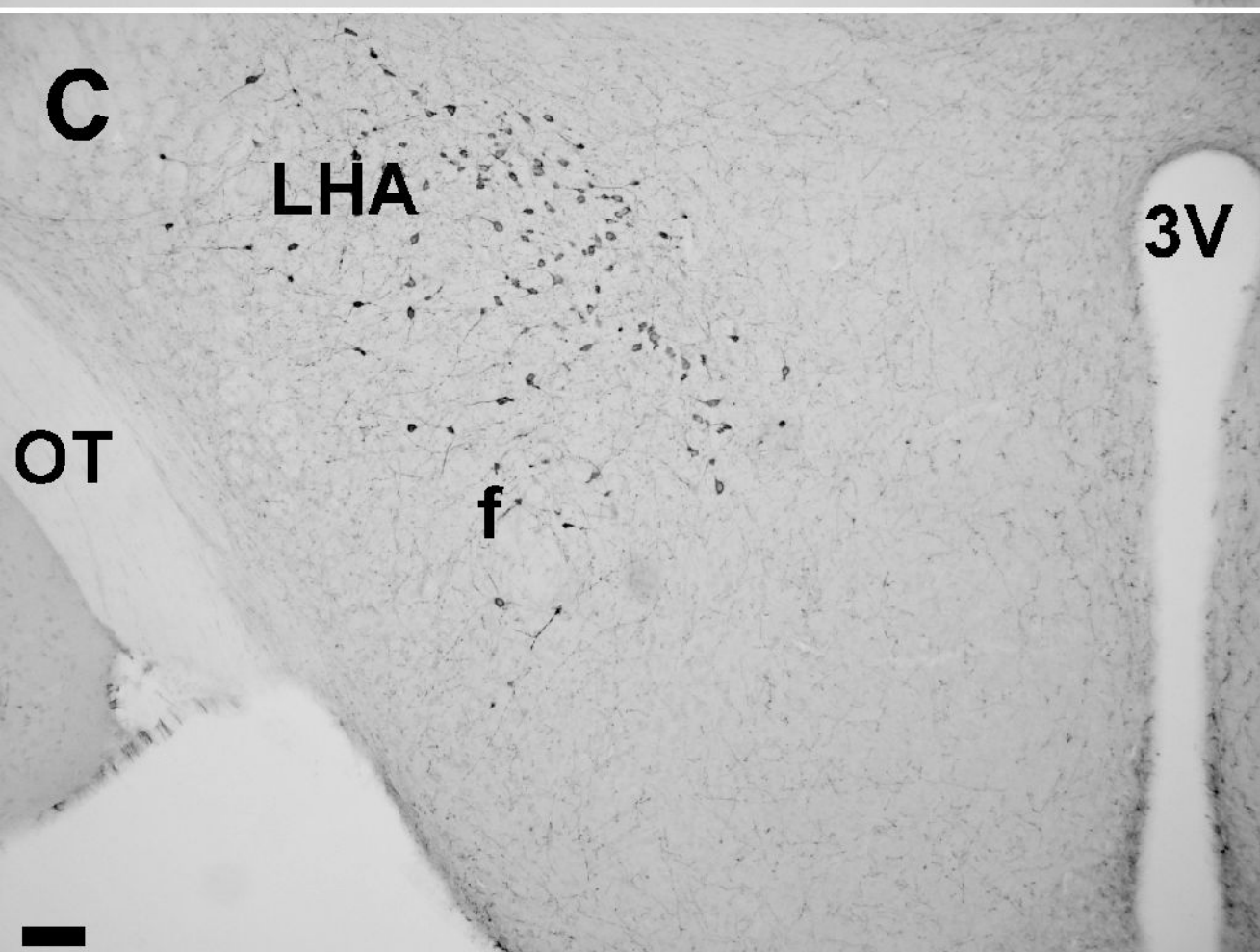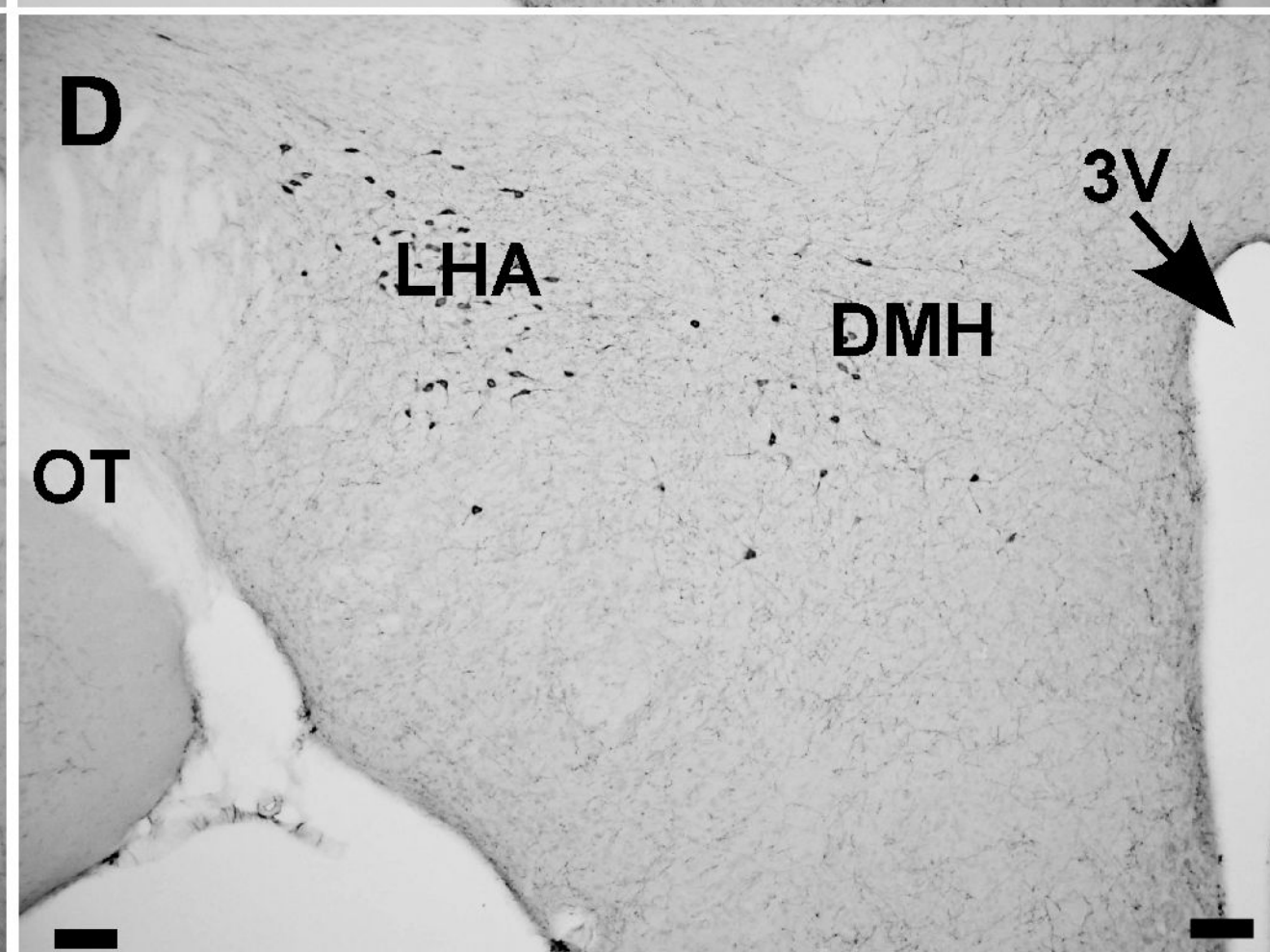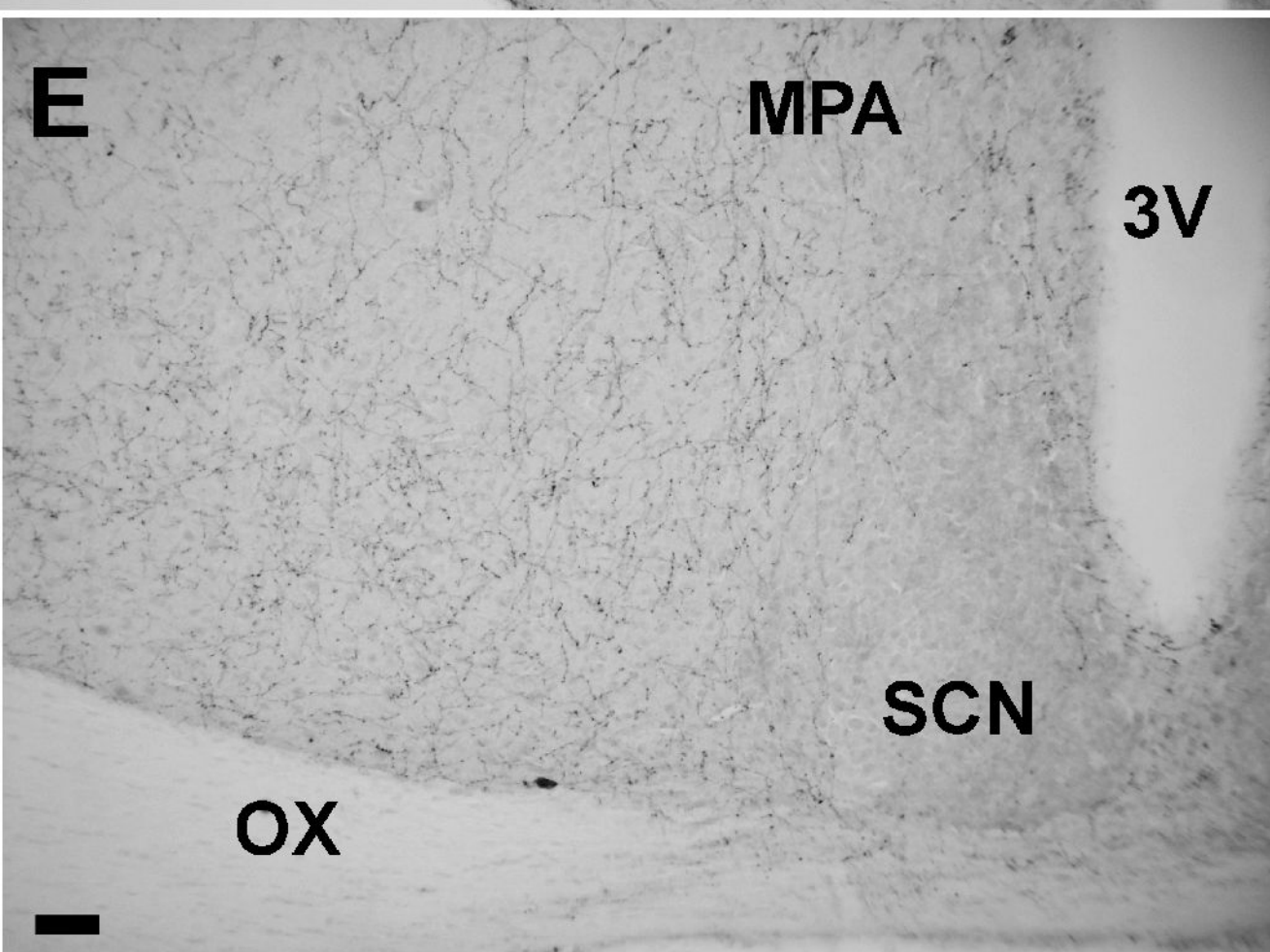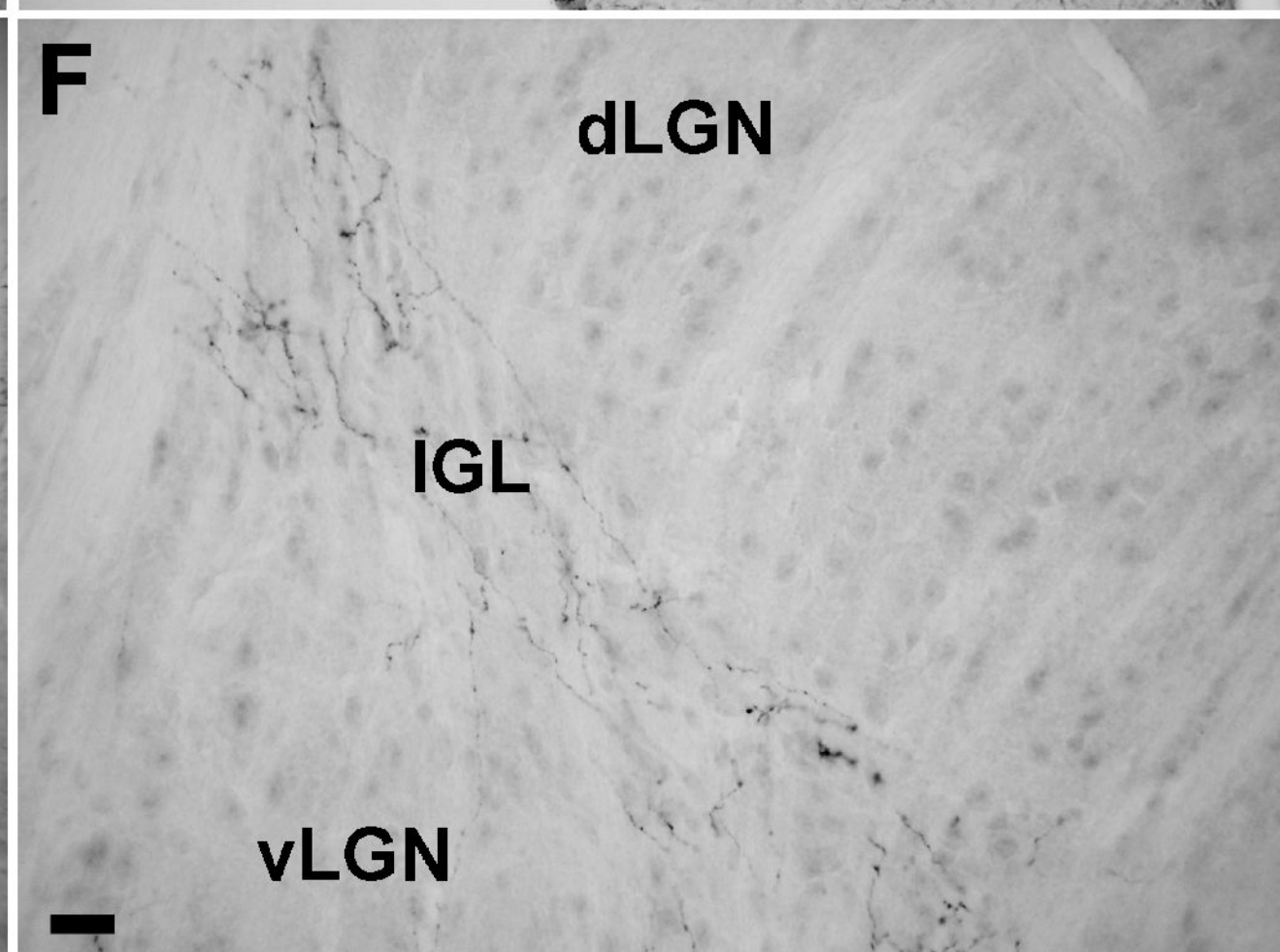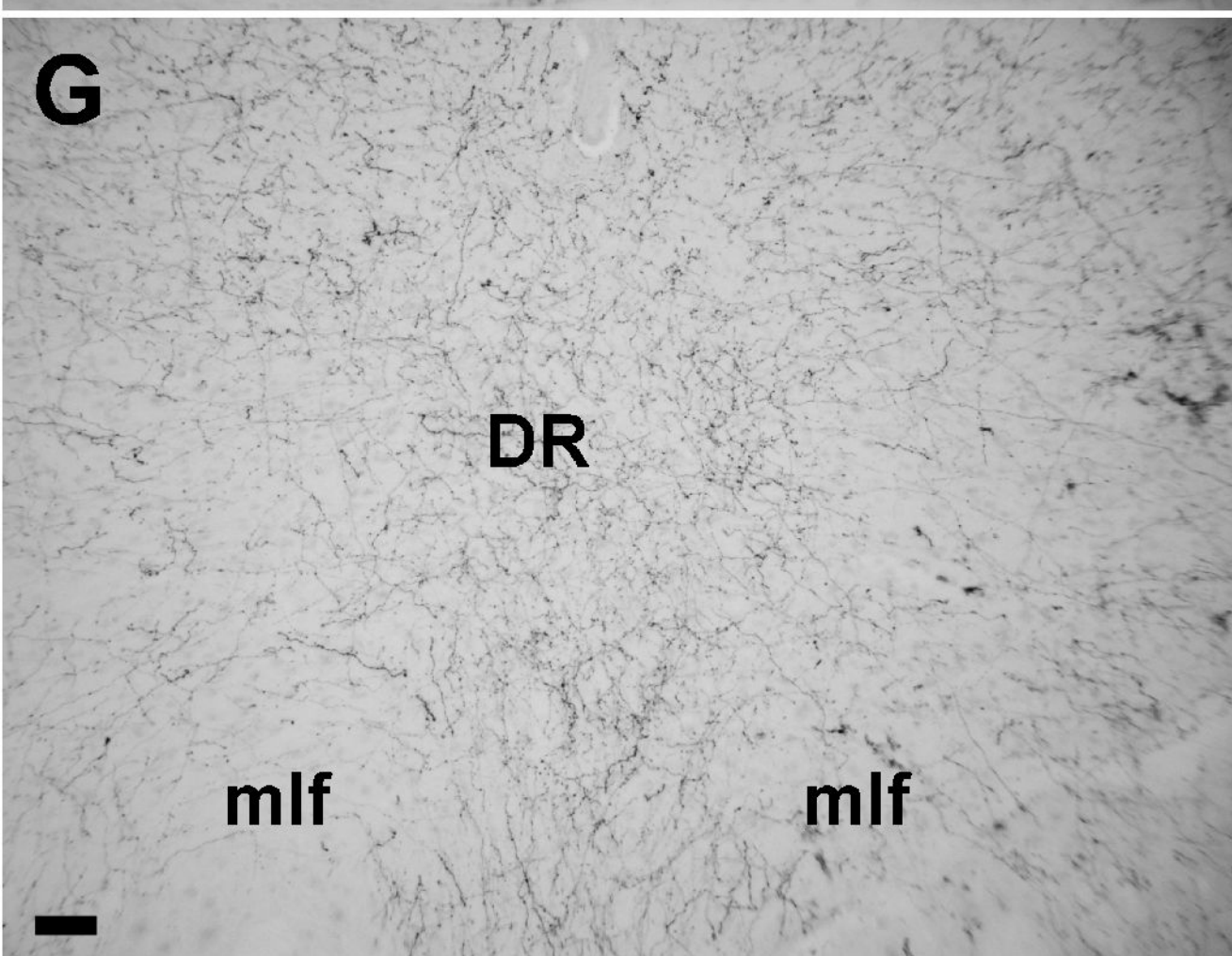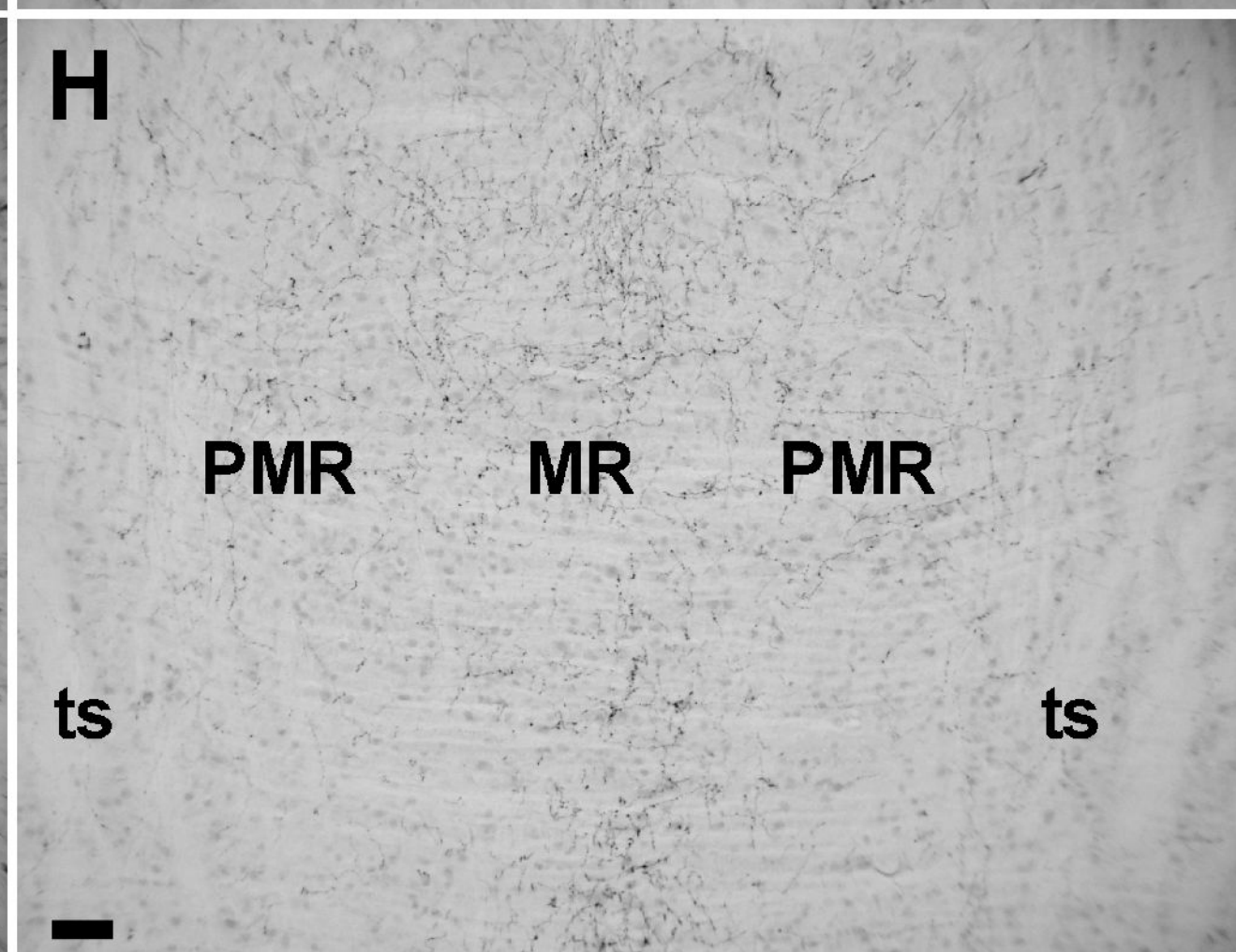

Supplement: Additional file 1 — Orexin-A-ir in the mouse hypothalamus, thalamus, and brainstem. Photomicrographs demonstrating regions with moderate to dense populations of orexin-A-ir neurons in the tuberal hypothalamus (arranged rostral to caudal, A to D) and orexin-A-ir fibres in structures of the circadian system including the suprachiasmatic nuclei (E), intergeniculate leaflet (F), dorsal raphe (G), and median raphe (H). 3 V = third ventricle; AHA = anterior hypothalamic area; DMH = dorsomedial hypothalamic area; DR = dorsal raphe nucleus; dLGN = dorsal lateral geniculate nucleus; f = fornix; IGL = intergeniculate leaflet; LHA = lateral hypothalamic area; mlf = medial longitudinal fasciculus; MPA = medial preoptic area; MR = median raphe nucleus; OX = optic tract; PMR = pontine median raphe nucleus; SCN = suprachiasmatic nucleus; TC = tuber cinereum; ts = tectospinal tract; vLGN = ventral lateral geniculate nucleus. Calibration bars = 100 μm (A-D), 50 μm (E, G, H), 25 μm (F). [file 1756-6606-1-19-S1.pdf]

Supplemental Figure 1--'Counting Boxes' for Medial and Lateral Tuberal Hypothalamus

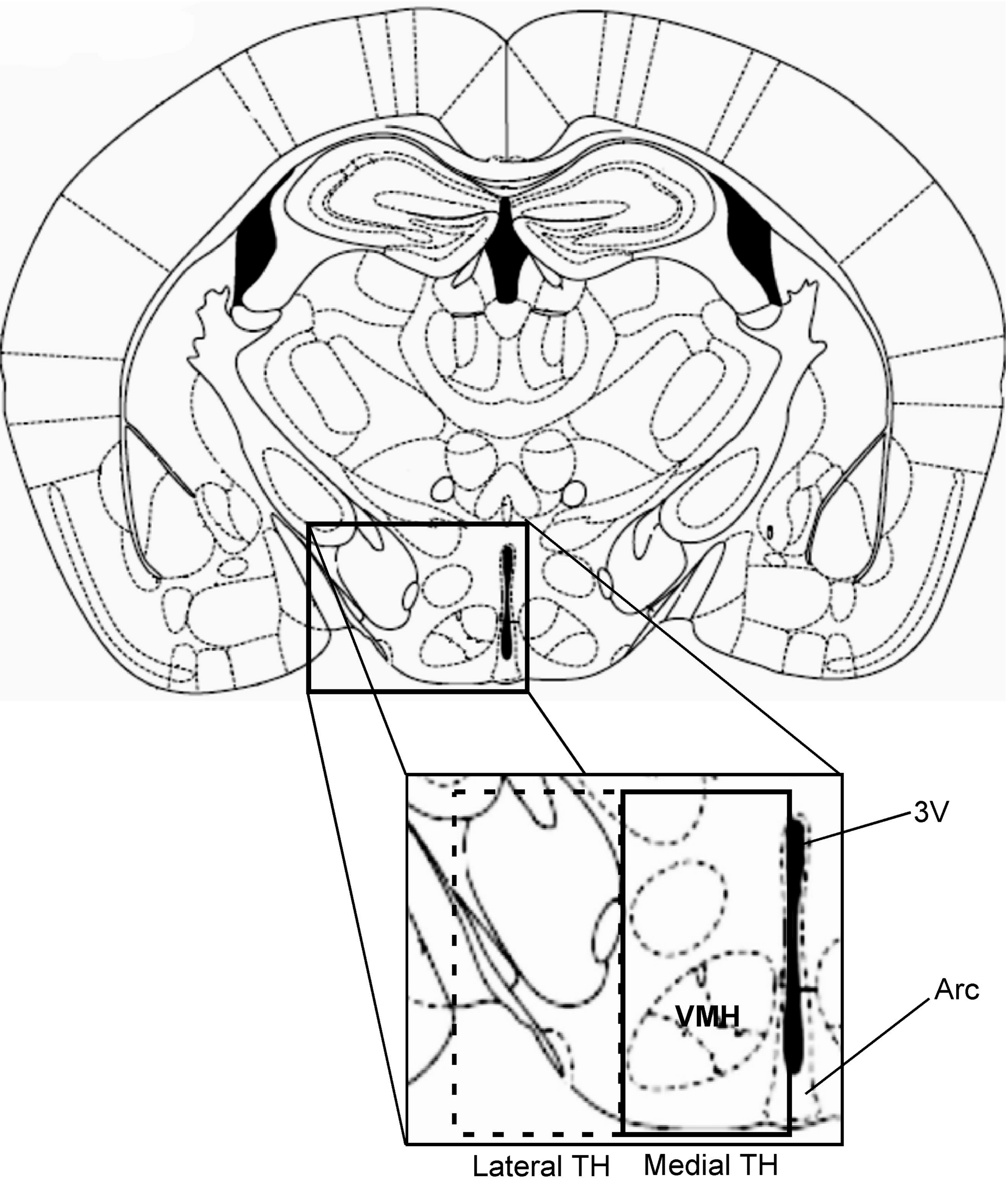

Supplement: Additional file 2 — Depicting the 'counting' boxes used to quantify c-Fos-ir and orexin-A-ir cells in the medial and lateral TH. Depiction of 'counting boxes' used to delineate the medial and lateral tuberal hypothalamus regions (at this level of the rostrocaudal axis) in which neurons single and double-labeled for c-Fos and orexin-A immunoreactivity were quantified. Image taken from Franklin and Paxinos (2001). VMH = ventromedial hypothalamus; 3 V = third ventricle; Arc = arcuate nucleus. [file 1756-6606-1-19-S2.pdf]
